# Physicochemical Properties of Wild Yam (*Dioscorea villosa*) Starch

**DOI:** 10.1155/2023/8868218

**Published:** 2023-09-29

**Authors:** Abraham Olasupo Oladebeye, Aderonke Adenike Oladebeye, Jacob Olalekan Arawande

**Affiliations:** ^1^Department of Science Laboratory Technology, University of Medical Sciences, Ondo, Nigeria; ^2^Department of Food Technology, Auchi Polytechnic, Auchi, Nigeria

## Abstract

Native starch extracted from wild yam (*Dioscorea villosa*) was evaluated for its intrinsic physicochemical properties. From the results, essential metals such as K, Ca, P, and Fe were detected along with some nonessential heavy metals below the WHO permissible limits. Bulk density was 0.13–0.63 g/mL. The water absorption capacity, oil absorption capacity, swelling power, and solubility of the starch were pH-responsive. Thermal profiles showed onset temperature, *T*_*o*_ (59.21 °C), peak temperature, *T*_*p*_ (60.22 °C), endset temperature, *T*_*c*_ (63.12 °C), gelatinization enthalpy, Δ*H*_*gel*_ (0.54 J/g), temperature range of gelatinization, *R* (3.91 °C), and peak height index, PHI (1.87 J/g °C). Exhibiting a crystallite size of 0.03 nm, absorption peaks of 15.3119°, 24.4120°, and 18.4170°, corresponding to interplanar d-spacings of 3.7500 Ǻ, 5.14000 Ǻ, and 4.954610 Ǻ, were obtained. Evidence of C–H at 1338.1 cm^−1^, C–O at 640.0 cm^−1^, C–H stretch at 2829.7 cm^−1^, and a strong and broad O–H group at 3291.2 cm^−1^ were obtained. The starch granules had low particle sizes, were homogeneous, and were aggregates of irregular shapes. At a lower pH (2-4), the wild yam starch studied could be a potential absorbent material in the production of disposable diapers and female napkins and as biodegradable films due to its high hydrophobicity at a high pH (8-12).

## 1. Introduction

Yam, a species in the genus *Dioscorea* (family, *Dioscoreacea*), is a major home meal in West Africa, especially Nigeria [1, 2]. Although yams play a significant role in ensuring food security [3], only seven out of the six hundred species are predominantly utilized in West Africa [4]. However, the most prominent inedible yam species is called wild yam (*Dioscorea villosa*).

Wild yam is a perennial crop with nonfleshy but dried, narrow, and crocked roots, bearing horizontal branches of long creeping runners [5]. Wild yam is also known as colic root, twining, and tuberous vine and is native to North America and Anina. Wild yam species contain diosgenin and have medicinal properties similar to those of other plants [5]. Due to their powerful antifungal properties, they have been traditionally used for the treatment of inflammation, muscle spasms, and asthma [6, 7].

The inedibility of wild yam species is due to the presence of antinutrients such as phenols, tannins, hydrogen cyanide, oxalate, amylase inhibitor activity, and trypsin inhibitor, which however, this can be eliminated through soaking, cooking, autoclaving, baking, and oil-frying, resulting in a good dietary source with high proportions of carbohydrate, fibres, essential amino acids, and low-level fats and protein [8–11].

Starch is a highly versatile biopolymer that is widely used in both food and nonfood applications due to its exceptional bioavailability, biocompatibility, biodegradability, and low cost. It is composed of amylose and amylopectin chains and is readily available in various plant storage organs such as roots, tubers, corms, cormels, and legume grains. The functionality of starch in industrial products is influenced by its source and intrinsic physicochemical properties, including chemical composition, functional properties, morphology, thermal properties, and rheological properties [12–14].

This research paper emphasizes the importance of analyzing the intrinsic physicochemical properties of wild yam starch to determine its potential for increased use as the demand for starch continues to grow.

## 2. Materials and Methods

### 2.1. Materials

Freshly harvested tubers of wild yam tubers of the wild yam species (*Dioscorea villosa*) were obtained from a farm in Etsako-West Local Government Area, Edo State, Nigeria. The tubers were identified by the herbarium curator of the University of Medical Sciences, Ondo, Nigeria, having voucher details, UNIMED PBTH No. 012, and were deposited in the herbarium of the university. The reagents used in this investigation were of Analar quality and were utilized in their original form. All the methods were carried out following local and international institutional guidelines.

### 2.2. Isolation of Starch

To extract the starch content from wild yam, the tubers were first washed with distilled water, then peeled, sliced, and homogenized at room temperature using a Rico blender. The resulting slurry was mixed with a 4% Na_2_S_2_O_3_ solution and filtered through a muslin bag. The filtrate was left to settle for five hours, allowing the wet starch cake to settle to the bottom before decanting. The wet cake was then washed with distilled water and decanted several times to obtain pure wet starch cake. The cake was then dried in aluminium foil at 50 °C for 24 h and stored in a transparent polythene film at 4 °C before analyses.

### 2.3. Determination of Mineral Compositions

The mineral composition of a starch sample was analyzed using an EDXRF spectrometer from Oxford Instrument, Supreme (×8000). After drying, grinding, and sieving, a 200 mg, 2.5 cm diameter pellet was formed with a pellet-pressing machine. The pellet was then exposed to radiation from a Cd-109 radioactive source for 2500 s while placed in the sample compartment. To correct for absorption, two measurements were taken using a liquid nitrogen-cooled Si (Li) detector. Different filters were used to ensure optimal detection of minerals [15, 16].

### 2.4. Determination of Bulk Density

The starch sample (1 g) was measured into a 25 mL graduated measuring cylinder and gently tapped on the bench top ten times from a height of 5 cm. After tapping, the volume of the sample was recorded. This process was repeated for 2, 3, 4, and 5 g weights of the same starch sample. Bulk density (BD) was calculated as the ratio of the weight of the sample to the volume of the sample after tapping [17].

### 2.5. Variation of Water and Oil Absorption Capacities with pH

One (1) g of the starch sample was mixed with 10 mL of distilled water/oil using a Thermolyne Maxi mix II mixer (type 37600) for 30 seconds to obtain a slurry. The pH of the slurry was then adjusted to 2, 4, 6, 8, 10, and 12 by adding 0.1 M HCl and 0.1 M NaOH while stirring. After allowing the slurries to stand at 21 °C for 30 min, they were centrifuged at 5000 rpm for 30 min, and the volume of the supernatant was measured in a 10 mL graduated cylinder. The results were expressed on a dry weight basis, using densities of 1 g/mL and 0.89 g/mL for distilled water and Devon King's Vegetable Oil, respectively [18, 19].

### 2.6. Variation of Swelling Power and Solubility with pH

Six preweighed test tubes were taken, and 0.1 g of starch was dispersed in 50 mL of distilled water in each one. The pH of each solution was then adjusted to 2, 4, 6, 8, 10, and 12 by adding 0.1 M HCl or 0.1 M NaOH while stirring. The resulting slurries were heated in a water bath at 90 °C for 30 min and then allowed to cool down to 28 ± 2 °C. After centrifuging the slurries at 2500 rpm for 15 minutes, the supernatants were poured off. Finally, the swelling power was calculated using a specific equation [20].

(1) $$\begin{array}{c} \text{Swelling power }\left(\text{g}/\text{g}\right)=\frac{\text{weight of the swollen starch sample}}{\text{weight of the native starch sample}}. \end{array}$$

For solubility, the supernatants were spread onto clean, preweighed Petri dishes and dried in an oven at 100 °C until a constant weight was achieved [20].

(2) $$\begin{array}{c} \text{Solubility }\left(\%\right)=\frac{\text{weight of the solubles}}{\text{weight of the sample}}\times 100. \end{array}$$

### 2.7. Thermal Properties

The thermal properties of the starch were analyzed using a differential scanning calorimeter (DSC-Q100, TA Instruments, New Castle, DE, USA). A sample of 3.00 ± 0.01 mg starch (mixed with water in a ratio of 1 : 3) was placed in an aluminium sample pan, which was then sealed and weighed. The pan was allowed to equilibrate at room temperature for an hour. The machine was conditioned at 75 °C for 2 hours, and the reference and indium pans were conditioned at a rate of 10 °C/min between 30-180 °C for 20 min. The sample was then tested for thermal properties by replacing the indium pan with the sample pans and heating at a rate of 5 °C/min from 20 to 100 °C. The DSC cell was purged with nitrogen gas at a flow rate of 100 mL/min, and the thermal profiles were generated using TA Instruments Universal Analysis software [21].

### 2.8. X-Ray Diffractometry

The starch sample was equilibrated in a desiccator for three days above distilled water. It was then subjected to X-ray diffraction analysis using a D-5000 Siemens diffractometer with Ni-filtered Cu-K*α* radiation. The resulting X-ray patterns were analyzed using peak positions to determine the starch sample's crystalline nature [4].

(3) $$\begin{array}{c} \text{Crystallite size},D\left(\text{hkl}\right)=\frac{k\lambda }{B_{\left(\text{hkl}\right)}\cos\theta }, \end{array}$$

where *k* is the Scherrer constant (0.84), *λ* is 0.154 nm, *B*_(hkl)_ = FWHM (Full Width Half Maximum), and *θ* is the corresponding Bragg's angle to FWHM.

### 2.9. FTIR Spectroscopy

The functional groups present in the starch sample were identified using a Nicolet AVATAR 360 Fourier infrared spectrometer with KBR disks. Prior to testing, the sample was pressed into a pellet and dispersed in a KBR matrix of 100 mg, with a total weight of 1.0 mg. A spectral resolution of 4 cm^−1^ was used, and 32 scans were recorded for the sample [20].

### 2.10. Morphology

Gold-coated starch granules were positioned on an aluminium specimen stub and observed using a scanning electron microscope (SEM-FEI model Q250, FEI Netherlands), which was equipped with a sputter coater (SC 515 VG Microtech, Sussex, UK). The sample morphology was analyzed at various magnifications, including 150 K, 500 K, and 1000 K [22].

### 2.11. Statistical Analysis

The statistical analysis of the data collected for mean comparison was conducted with SPSS 26.0 software. Duncan's least significant test and one-way analysis of variance (ANOVA) were utilized at a significance level of 5%.

## 3. Results and Discussion

### 3.1. Mineral Compositions

Bioaccumulation of K, Ca, P, Fe, Cr, S, Al, Si, Cl, Ti, and Mn in wild yam starch was studied (Table 1). Fe, Cr, Al, Ti, and Mn are the heavy metals found in the wild yam starch. The bioaccumulation of metals in plants usually depends on their bioavailability in the soil, the soil type, pH, and time of harvest [23]. Although there are no WHO permissible limits for the concentrations of Al and Ti, the concentrations of the other heavy metals obtained in this study are well below the recommended limits by the WHO [24]. Studies suggest that Ti is a complementary element to Fe when it comes to plant growth [25]. After analyzing wild yam starch, it was found that the most abundant element present was silicon (32.87 ± 0.15 mg/kg), followed by potassium (7.98 ± 0.12 mg/kg). The swelling power of starch is affected by the concentration of phosphorus; a higher phosphorus content results in a higher swelling power of starch [26]. Wild yam starch also contains chlorine (0.89 ± 0.02 mg/kg), which is important for medicinal purposes. Chlorine is the second most abundant electrolyte in serum after sodium and plays a crucial role in facilitating homeostasis, electrical neutrality, the acid-base buffering system, and assessing various pathological conditions [27]. The wild yam starch studied contains a 2.12 ± 0.01 mg/kg concentration of sulphur, which might indicate the presence of sulphur-containing amino acids in the starch studied [28, 29].

### 3.2. Bulk Density

The bulk density is in a constant ratio of 1 : 1 with the weight of the starch sample studied (Table 2). Thus, irrespective of the weight of the starch used, the bulk density remains the same. The bulk density of starch has been adduced to the presence of amylose, which increases as amylose content increases up to 50% before decreasing. Starch with low bulk density is a suitable recipe for snack foods, which favours consumer acceptability in terms of crispiness, softness, and mouthfeel [30].

### 3.3. Variation of Water and Oil Absorption Capacities with pH

**Figure 1:**
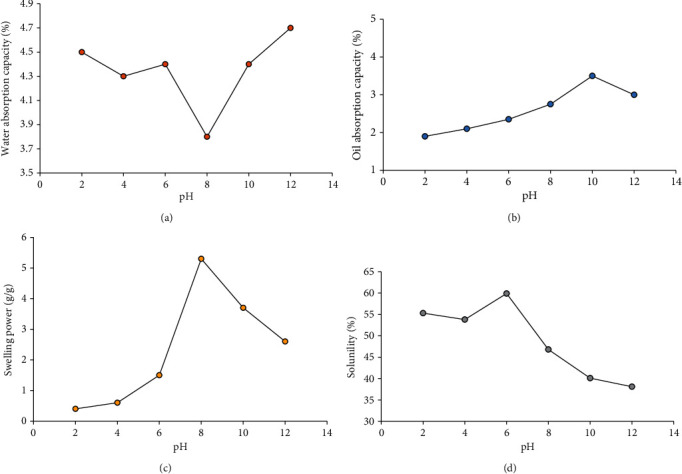
Variation of pH with (a) WAC, (b) OAC, (c) swelling power, and (d) solubility of wild yam starch.

The water and oil absorption capacities of wild yam starch studied are pH-responsive, showing significantly different (*p* < 0.05) values as pH changes from 2 to 12 (Figures 1(a) and 1(b)). A general reduction in water absorption capacity (WAC) as pH increases from 2 to 8 and a general elevation in WAC as pH increases from 8 to 12 are observed. An anomalous exception to this trend is observed at pH 6. A peak WAC of 4.70 ± 0.01% is obtained at pH 12. The WAC of starch gives information on the number of water molecules available in the starch interstitial network, which will aid in gelatinization. The more acidic the medium, the more structured the starch granules and the less water molecules are bound to the starch molecule network; thus, the starch chain becomes more hydrophobic, and dehydration of the starch granules is allowed [31, 32]. This phenomenon is observed until a change occurs as the pH increases from 8, presenting hydrophilic and more hydrated starch granules as the medium becomes more alkaline, which changes the starch structure from crystalline to amorphous and allows more binding sites for water molecules. High WAC is desirable for optimal gelatinization.

From this study, a peak oil absorption capacity (OAC) of 3.50 ± 0.01% is obtained at pH 10. As the pH increases from 2 to 10, OAC increases because the intermolecular bonds of the starch granules are weak; thus, the pores are enlarged, oil molecules are absorbed and retained in the interstitial network, and the starch granules behave oleophilic in nature [33]. The implication of this observation is the tendency of the starch studied to inhibit gel formation between pH 2 and 10, which is evident, to a high degree, in the results obtained for its WAC. High OAC is desirable in starch-based food emulsifiers and products such as mayonnaise.

### 3.4. Variation of Swelling Power and Solubility with pH

Figures 1(c) and 1(d) present the swelling and solubility profiles of native wild yam (*Dioscorea villosa*) starch studied at different pH of 2, 4, 6, 8, 10, and 12. The swelling and solubility of wild yam starch are pH-sensitive and vary significantly at different pH levels (*p* < 0.05). Swelling power (SP) is an indicator of the ability of the starch to hold water and form gels at temperatures close to or equal to its gelatinization temperature [32]. The SP of the starch increases as the pH changes from acidic to alkaline (between 2 and 8), followed by a gradual decrease after pH 8. At high gelatinization temperatures, the amylose and amylopectin molecules in the starch become more entangled, leading to the formation of a gel. This is due to an increase in the crystalline region and a decrease in the amorphous region. As a result, the starch network becomes more hydrophilic [31, 32]. Starch performs better in an acidic or slightly acidic environment. The decline in swelling power as pH increases may be attributed to the potential loss of amylose molecules from the swollen starch granules [34]. Wild yam starch-based fluids produced at pH 8 have no negative effects on human health when used for diagnoses such as magnetic resonance imaging (MRI) and magnetic particle imaging (MPI). The solubility of native wild yam starch varies as pH increases from 2 to 12. Its solubility decreases from 55.30% at pH 2 to 53.80% at pH 4, before peaking at 59.90% at pH 6, and then gradually decreasing as the alkalinity increases. Starch granules usually swell and disintegrate to release soluble materials, including amylose molecules [35]. As the degree of alkalinity increases, the likelihood of wild yam starch granules forming a complex with protein decreases. However, starch stability improves due to starch-protein interaction because soluble proteins are more likely to reside in phase with hydrophilic starch [36]. This phenomenon is not favoured as alkalinity increases. The decreased solubility of starch is due to the entanglement between amylose and amylopectin molecules, which favours the formation of a starch gel [37].

### 3.5. Thermal Properties

The onset temperature (*T*_*o*_), peak temperature (*T*_*p*_), and endset temperature (*T*_*c*_) of gelatinization of the wild yam starch are 59.21, 60.22, and 63.12 °C, respectively (Table 3). The value of *T*_*o*_ obtained for wild yam starch is in league with that value reported for tapioca (59.8 °C) [38]. The stability of starch when exposed to heat is affected by its surface area, which is determined by its particle size. The smaller the size of the particles, the more stable the starch will be [39]. This may also account for a low gelatinization enthalpy (Δ*H*_*gel*_) of 0.54 J/g compared to 17.0 J/g reported for potato and taro [40, 41]. The temperature range of gelatinization (*R*) obtained for the starch sample is 3.91 °C. *R* is the difference between *T*_*o*_ and *T*_*c*_ and significantly indicates the homogeneity or heterogeneity of starch granules. Homogeneity increases as *R* decreases [42]. The peak height index (PHI) of the sample (1.87 J/g °C) is low, and this may be a pointer to the high homogeneity of the starch granular architecture.

### 3.6. X-Ray Diffractometry

The three major peaks used in characterizing the wild yam starch sample show absorption bands at 15.3119°, 24.4120°, and 18.4170°, which correspond to the interplanar d-spacings of 3.7500 Ǻ, 5.14000 Ǻ, and 4.954610 Ǻ, respectively (Table 4). The data on absorption bands and interplanar d-spacing suggest that the wild yam starch studied is a C_B_-type starch [43]. The crystallite size of wild yam starch is 0.03 nm, indicating low particle size, high surface area, and thus high reactivity of the granules [44]. Highly reactive starch granules enable treatment agents to penetrate their networks, leading to better physicochemical properties of the starch for a variety of applications.

### 3.7. FTIR Pattern

**Figure 2:**
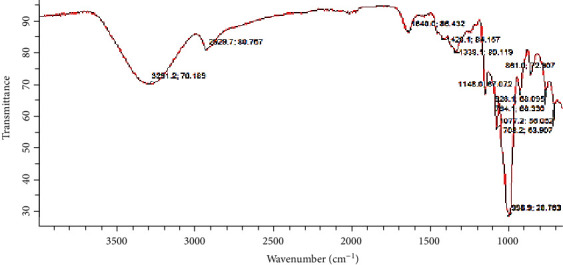
FTIR pattern of wild yam starch.

The FTIR pattern of wild yam (*Discorea villosa*) as a function of transmittance and wave number is depicted in Figure 2. The starch sample exhibits absorption peaks at 708.21, 784.1, 961.0, 928.1, 998.9, 1077.2, 1148.0, 1338.1, 1420.1, 1640.0, 2829.7, and 3291.2 cm^−1^ corresponding to transmittance values of 63.907, 68.330, 72.907, 68.095, 28.763, 56.052, 67.672, 80.119, 84.157, 86.432, 80.757, and 70.189%, respectively. The fingerprint representations at 928.1 cm^−1^ and 998.9 cm^−1^ are evidence of the presence of vinyl C–H out-of-plane bending in the starch sample studied. The transmittance peak at 1338.1 cm^−1^ represents CH in-plane bending, 1640.0 cm^−1^ is the carbonyl group, C–O, which can be oxidized to a carboxyl group, 2829.7 cm^−1^ is the C–H stretch of alkanes, and 3291.2 cm^−1^ represents a strong and broad O–H group, which is indicative of a strong hydrogen bonding between the starch molecules [45–47]. The swelling power of starches is facilitated by a strong hydrogen bond, which also inhibits their solubility [47, 48]. This unique feature enables wild yam starch to form a gel in an acidic or neutral medium with a pH of 7 or less when exposed to high temperatures.

### 3.8. Morphology

**Figure 3:**
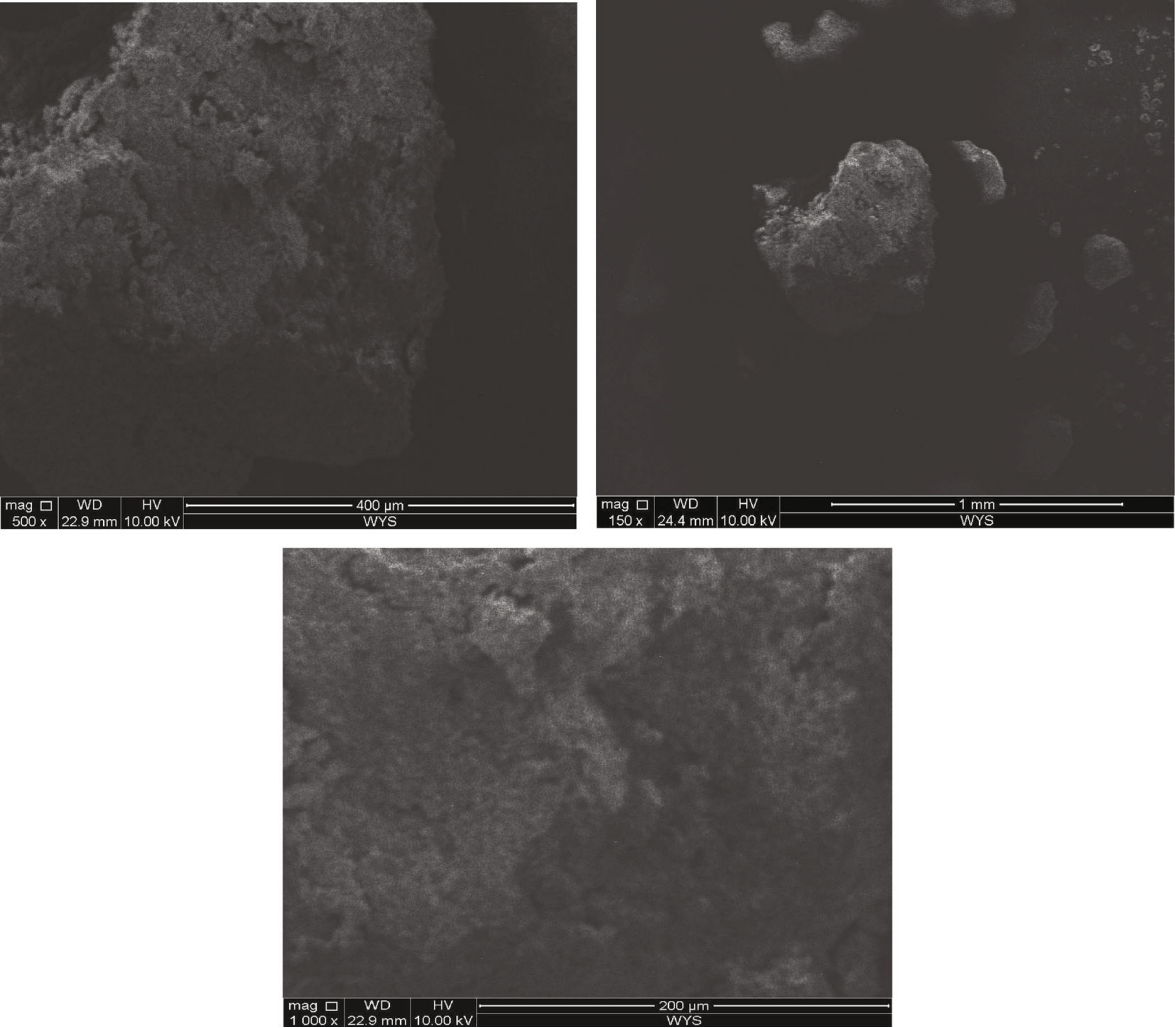
Microstructures of wild yam starch at different magnifications.

As shown in Figure 3, the morphological study of the starch sample shows that the granules have irregular shapes and are aggregated. The granules are generally homogeneous, which could be due to their small particle size. This observation is consistent with the thermal characteristics of the starch in terms of the range of gelatinization temperature (*R*) and PHI, as previously reported in studies [14, 21]. There were no visible pores or damages on the surface of the granules. It is worth noting that any pores or surface damage to starch granules could be a result of the process of starch isolation, which may distort their architectural structure if not handled properly, as reported in previous studies [14, 22].

## 4. Conclusion

The native starch isolated from wild yam (*Discorea villosa*) exhibits intrinsic physicochemical properties. The wild yam starch studied bioaccumulates some macro and heavy metals (K, Ca, P, Fe, Cr, S, Al, Si, Cl, Ti, and Mn) in nonlethal proportions. The bulk density is not altered by a change in the weight of the starch sample, whereas the water absorption capacity, oil absorption capacity, swelling power, and solubility are pH-responsive. Swelling power is elevated in an acidic medium, while solubility is elevated in an alkaline medium. The granules of wild yam starch are thermally stable and homogeneous. Strong hydrogen bonding is identified between the molecules of the starch granules. Due to its intrinsic properties, the wild yam starch studied can serve as a suitable recipe for snack foods, food emulsifiers, and products such as mayonnaise. Its crystallite size is advantageous where penetration of the treatment agent into a starch matrix is required. At a lower pH (2-4), the wild yam starch studied could be a potential absorbent material in the production of disposable diapers and female napkins and as biodegradable films due to its high hydrophobicity at a high pH (8-12). At a pH level of 8, which is similar to the natural pH level of the body, wild yam starch is safe for human use as a diagnostic fluid in MRI and MPI recipes. The granules are shaped irregularly but are arranged in separate clusters.

## Figures and Tables

**Table 1:** Elemental compositions of wild yam starch.

Element	K	Ca	P	Fe	Cr	S	Cl	Al	Si	Ti	Mn
Concentration (mg/kg)	7.98^b^ ± 0.12	1.05^g^ ± 0.00	1.27^f^ ± 0.01	1.66^e^ ± 0.02	0.03^k^ ± 0.00	2.12^d^ ± 0.01	0.89^h^ ± 0.02	5.21^c^ ± 0.01	32.87^a^ ± 0.15	0.08^j^ ± 0.00	0.10^i^ ± 0.00

Results are expressed as *means* ± *standard* deviations (*n* = 3). Values in the same row with the same superscript letters (a > b > c > d > e > f > g > h > i > j > k) are not significantly different (*p* < 0.05).

**Table 2:** Bulk density of wild yam starch.

Property	Sample weight (g)				
	1.00	2.00	3.00	4.00	5.00
Bulk density (g/mL)	0.13^a^ ± 0.01	0.25^b^ ± 0.01	0.38^c^ ± 0.01	0.50^d^ ± 0.01	0.63^e^ ± 0.01

Results are expressed as means ± standard deviations (*n* = 3). Values in the same row with the same superscript letters (a > b > c > d > e) are not significantly different (*p* < 0.05).

**Table 3:** Thermal properties of wild yam starch.

*T*_*o*_ (°C)	*T*_*p*_ (°C)	*T*_*e*_ (°C)	Δ*H*_gel_ (J/g)	*R* (°C)	PHI (J/g °C)
59.21	60.22	63.12	0.54	3.91	1.87

**Table 4:** Major peaks of X-ray diffractogram of wild yam starch.

Peak 1			Peak 2			Peak 3			Crystallite size (nm)
2*θ*	d (Å)	I (counts)	2*θ*	d (Å)	I (counts)	2*θ*	d (Å)	I (counts)	
15.31	3.75	50	24.41	5.14	70	8.42	4.95	100	0.03

## Data Availability

The data that support the findings of this study are available on request from the corresponding author. The data are not publicly available due to privacy or ethical restrictions.

## Additional Points

*Herbarium Voucher Label*. The authors have permission to collect wild yam (*Dioscorea villosa*) tubers for research purposes with voucher details, UNIMED PBTH No. 012. The voucher specimens were identified by Miss Jumoke Olofinlade (Herbarium Curator, University of Medical Sciences, Ondo, Nigeria), and the specimens were deposited in the herbarium of the university.
